# A Conserved Supergene Locus Controls Colour Pattern Diversity in *Heliconius* Butterflies

**DOI:** 10.1371/journal.pbio.0040303

**Published:** 2006-09-26

**Authors:** Mathieu Joron, Riccardo Papa, Margarita Beltrán, Nicola Chamberlain, Jesús Mavárez, Simon Baxter, Moisés Abanto, Eldredge Bermingham, Sean J Humphray, Jane Rogers, Helen Beasley, Karen Barlow, Richard H. ffrench-Constant, James Mallet, W. Owen McMillan, Chris D Jiggins

**Affiliations:** 1 Institute of Evolutionary Biology, School of Biological Sciences, University of Edinburgh, Edinburgh, United Kingdom; 2 Institute of Biology, Leiden University, Leiden, Netherlands; 3 The Galton Laboratory, Department of Biology, University College London, London, United Kingdom; 4 Department of Biology, University of Puerto Rico, San Juan, Puerto Rico; 5 Department of Biology and Biochemistry, University of Bath, Bath, United Kingdom; 6 Smithsonian Tropical Research Institute, Balboa, Panama; 7 Jirón Alegría Arias de Morey, Tarapoto, Peru; 8 The Wellcome Trust Sanger Institute, Cambridge, United Kingdom; Duke University, United States of America

## Abstract

We studied whether similar developmental genetic mechanisms are involved in both convergent and divergent evolution. Mimetic insects are known for their diversity of patterns as well as their remarkable evolutionary convergence, and they have played an important role in controversies over the respective roles of selection and constraints in adaptive evolution. Here we contrast three butterfly species, all classic examples of Müllerian mimicry. We used a genetic linkage map to show that a locus, *Yb,* which controls the presence of a yellow band in geographic races of *Heliconius melpomene,* maps precisely to the same location as the locus *Cr,* which has very similar phenotypic effects in its co-mimic H. erato. Furthermore, the same genomic location acts as a “supergene”, determining multiple sympatric morphs in a third species, *H. numata. H. numata* is a species with a very different phenotypic appearance, whose many forms mimic different unrelated ithomiine butterflies in the genus *Melinaea*. Other unlinked colour pattern loci map to a homologous linkage group in the co-mimics H. melpomene and *H. erato,* but they are not involved in mimetic polymorphism in H. numata. Hence, a single region from the multilocus colour pattern architecture of H. melpomene and H. erato appears to have gained control of the entire wing-pattern variability in *H. numata,* presumably as a result of selection for mimetic “supergene” polymorphism without intermediates. Although we cannot at this stage confirm the homology of the loci segregating in the three species, our results imply that a conserved yet relatively unconstrained mechanism underlying pattern switching can affect mimicry in radically different ways. We also show that adaptive evolution, both convergent and diversifying, can occur by the repeated involvement of the same genomic regions.

## Abstract 2

In an intriguing example of adaptive evolution, genetic linkage analysis identifies a conserved region in distantly related*Heliconius* butterfly species that controls the diverse effects of wing patterning and mimicry.

## Introduction

Recent interest has focused on the genetic basis of convergent evolution [1,2]. Adaptive convergence between unrelated species, exemplified by colour pattern mimicry in insects [3], has led to a long-standing controversy about the relative contribution of gradual evolution driven by natural selection [4] versus occasional phenotypic leaps facilitated by conserved developmental pathways [5]. Recently, molecular genetic studies have shed new light on this controversy and have shown that regulation of the same genes [6,7], or even repeated recruitment of the same alleles [8], may explain convergent phenotypes in nature.

However, analysis of convergent phenotypes is only part of the story, because convergence and parallelism commonly occur in groups of organisms that have undergone recent adaptive radiations [9–11]. We are therefore interested in the evolution of phenotypic diversity and whether similar developmental genetic mechanisms are involved in convergent and divergent evolution. The repeated involvement of homologous loci in the evolution of convergent phenotypes would appear to support a hypothesis of strong developmental constraints on adaptive evolution [11–13]. If the same loci are also recruited in divergent evolution, then they may be generally important in phenotypic evolution rather than solely playing a role in convergence [14].

With strong divergence between geographic races of the same species and near-perfect local mimetic convergence between species, the diverse wing patterns of *Heliconius* butterflies (Nymphalidae: Heliconiinae) provide an opportunity to link molecular genetics to adaptive evolution. A few genes of major effect are known to control patterns in the Müllerian co-mimics H. erato and H. melpomene [15]*.* This has led to proposals that homologous genetic pathways [16] or a limited number of loci capable of controlling colour pattern shifts [17] could play an important role in convergent mimicry. However, homology of genetic architecture in mimetic butterflies has never been directly tested, despite the key role that mimicry has played in the history of the controversy [4,5].

**Figure 1:**
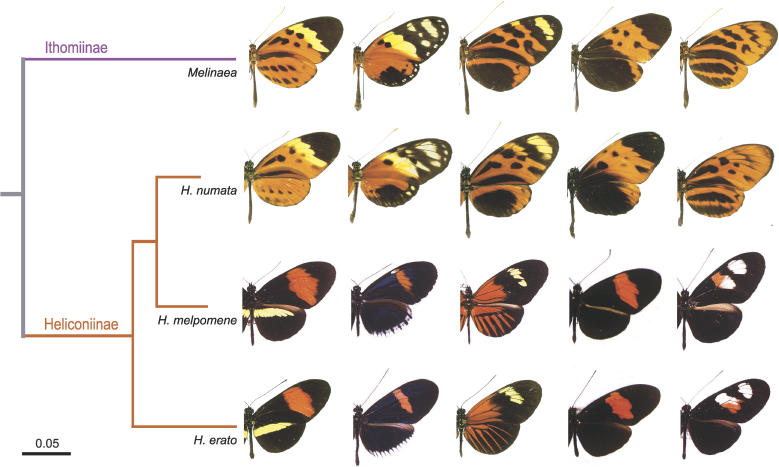
Colour Pattern Diversity of *H. numata, H. melpomene,* and their Respective Co-Mimics The upper half of the figure shows five sympatric forms of H. numata from northern Peru (second row, left to right: *H. n.* f. *tarapotensis, H. n.* f. *silvana, H. n.* f. *aurora, H. n.* f. *bicoloratus,* and *H. n.* f. *arcuella*) with their distantly related comimetic *Melinaea* species (Nymphalidae: Ithomiinae) from the same area (first row: M. menophilus ssp. nov*.*, *M. ludovica ludovica, M. marsaeus rileyi, M. marsaeus mothone,* and *M. marsaeus phasiana*) [20]. The lower half of the figure shows five colour pattern races of *H. melpomene,* each from a different area of South America (third row: *H. m. rosina, H. m. cythera, H. m. aglaope, H. m. melpomene,* and *H. m. plesseni*) with their distantly related comimetic H. erato races from the same areas (fourth row: *H. e.* cf. *petiveranus, H. e. cyrbia, H. e. emma, H. e. hydara,* and *H. e. notabilis*). *H. m. aglaope* and *H. e. emma* are known as rayed forms, whereas *H. m. rosina, H. m. melpomene,* and co-mimics are known as postman forms. H. melpomene and H. erato are from divergent clades of *Heliconius* and are identified in the field using minor morphological characters, such as the different form of the red rays on the hindwing between *H. m. aglaope* and *H. e. emma* (third from left) or the arrangement of red versus white patches in *H. m. plesseni* and *H. e. notabilis* (first from right). Co-mimics H. numata and Melinaea spp. belong to different subfamilies of the Nymphalidae and have very different body morphology and wing venation. The phylogram on the left is a maximum-likelihood tree based on 1,541 bases of mitochondrial DNA (scale bar in substitutions per site, all bootstrap values over 99).

We investigated the genetic architecture of colour pattern in three *Heliconius* species that represent examples of both mimetic convergence and colour pattern diversification. H. melpomene and H. erato are distantly related, yet are phenotypically identical and have undergone a parallel radiation into over 30 named “rayed” or “postman” colour pattern races across the neotropics (Figure 1)*. H. erato* is the probable model for this radiation [18], and local populations of the two co-mimics are monomorphic. The third species, *H. numata,* is closely related to H. melpomene but has extremely divergent wing patterns. Unlike the patterns in H. melpomene or *H. erato,* these patterns are highly polymorphic within populations, with up to seven “tiger”-patterned morphs in a single locality [20,21] (Figure 1). Each of these morphs is a precise mimic of a different species of *Melinaea* (Nymphalidae: Ithomiinae); polymorphism in H. numata is thought to be maintained by strong selection for mimicry in a fine-scale spatial mosaic of ithomiine communities [19,20].

**Figure 2:**
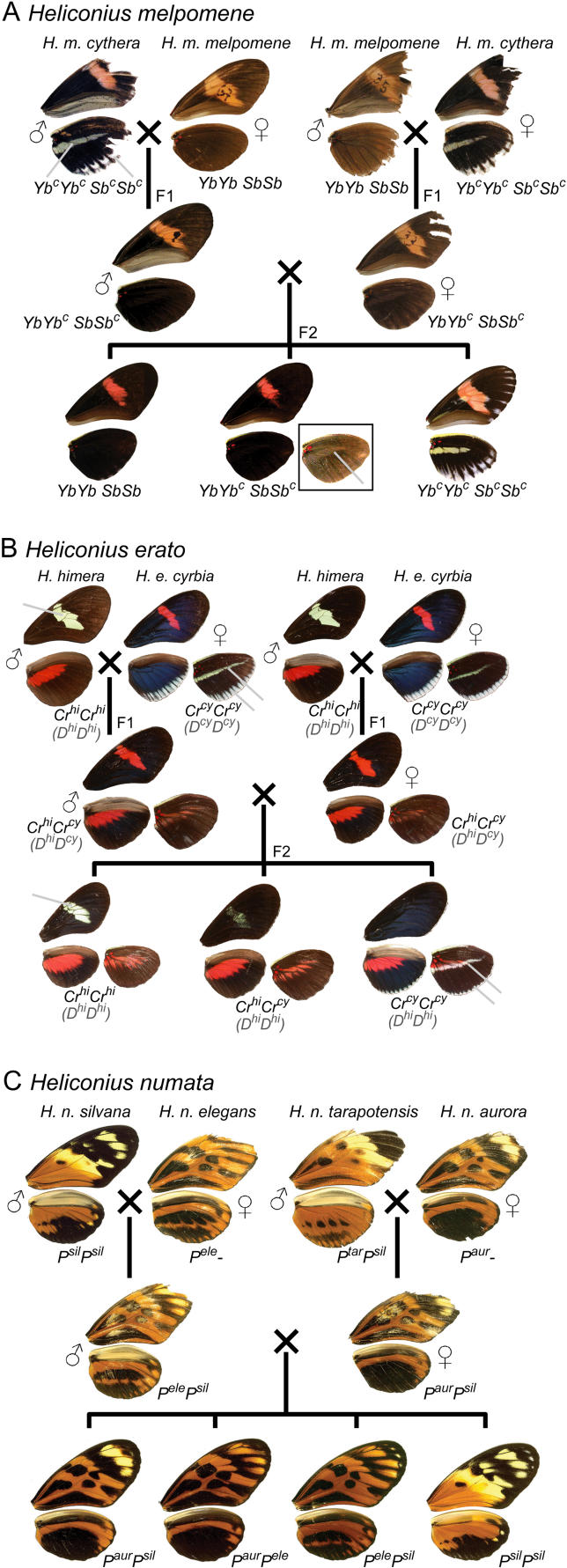
Crosses Used for Mapping the *Yb, P,* and *Cr* Loci (A) Crossing scheme in H. melpomene showing segregation of tightly linked loci *Yb* and *Sb* (hindwing yellow bar and white margin, present in *H. m. cythera, Yb^c^Yb^c^ Sb^c^Sb^c^*) in brood B033. Genotypes are shown on the figure*.* The hindwing image in the box has been manipulated to highlight the shadow hindwing bar characteristic of heterozygote genotypes. Segregation of the linked *N* locus controlling the yellow forewing band was followed in a different set of crosses not shown here (Table S1; Materials and Methods). (B) Crossing scheme in H. erato showing segregation of *Cr* alleles in brood CH-CH5; *Cr* controls the forewing yellow band (absent in *H. e. cyrbia, Cr^c^Cr^c^*), and the hindwing yellow bar and white margin (present in *H. e. cyrbia*). The red-patterning gene *D* also segregates in this cross, but is unlinked to *Cr;* only progeny with a *D^hi^D^hi^* genotype are shown on the figure (Table S2; see also [24] for a figure of a similar cross showing all nine possible genotypes). (C) Crossing scheme in H. numata showing segregation of the *P* alleles in intercross B502. F1 parents are heterozygous for different alleles, thus producing four different genotypes in the progeny. *P* switches the entire colour pattern, with strong dominance between sympatric alleles. Other broods (not shown) segregating for the very same *P^ele^* and *P^sil^* alleles were sired by the same male or its full brother (Table S3).

The differences in colour pattern between races of H. melpomene and H. erato are controlled by several Mendelian factors of large phenotypic effect [15,17]. In *H. melpomene,* a complex of at least three tightly linked loci *(N, Yb,* and *Sb)* control most of the variation in yellow and white pattern elements (Figures 1 and 2A), and recombination between these loci suggests that they lie just a few centimorgans (cM) apart [15,17,21]. Another pair of loci *(B* and *D),* situated on a different linkage group, controls most of the variation in the red pattern elements and interacts with *N* to control the colour of the forewing band [15,17] (Figure 1). Locus *Ac* controls the presence of a yellow patch in the discal cell of the forewing in some crosses [22]. Finally, locus *K,* unlinked to *N–Yb–Sb* or *B–D,* turns white patches to yellow in crosses between H. melpomene and H. cydno [21,23] (Table S1).

The radiation in H. erato has a similar genetic architecture, with a locus *Cr* that has similar phenotypic effects to the combined action of *N, Yb,* and *Sb* in H. melpomene. In crosses between *H. e. cyrbia* and a sister species, *H. himera, Cr* controls a hindwing yellow bar (cf. *Yb*), a white hindwing margin (cf. *Sb*) and the yellow forewing band of H. himera (cf. *N*) [24] (Figure 2B). Nonetheless, there are differences between the species: in inter-racial H. erato crosses the forewing yellow band is controlled by an unlinked locus, *D,* rather than by *Cr* [17]. *D* also controls most of the variation in the red pattern elements in a way that is analogous to the *B–D* complex in H. melpomene.

In contrast, mimicry polymorphism affecting yellow, brown/orange, and black colour patterns in H. numata is inherited entirely at a single Mendelian locus, *P* (Figure 2C). Populations are locally polymorphic, and nine distinctive alleles have been identified for the *P* locus in a narrow geographic area of Peru (Figures 1 and 2C) [19,20]. Alleles at the *P* locus are nearly all completely dominant, with a linear hierarchy of dominance relationships [19,20], as might be expected in order to prevent the segregation of intermediate and nonmimetic phenotypes in wild populations. Occasional recombinant phenotypes occur, suggesting that the *P* locus may be a tight cluster of genes, or “supergene” [19,25].

Despite suggestions in the literature that there might be genetic homology between some of these mimicry genes in different *Heliconius* species [16,26,27], such homology has not been directly tested. Here we describe the development of molecular markers that are tightly linked to a colour pattern locus in *H. melpomene;* we used these markers to investigate synteny and homology of colour pattern genes between the three *Heliconius* species.

## Results

**Figure 3:**
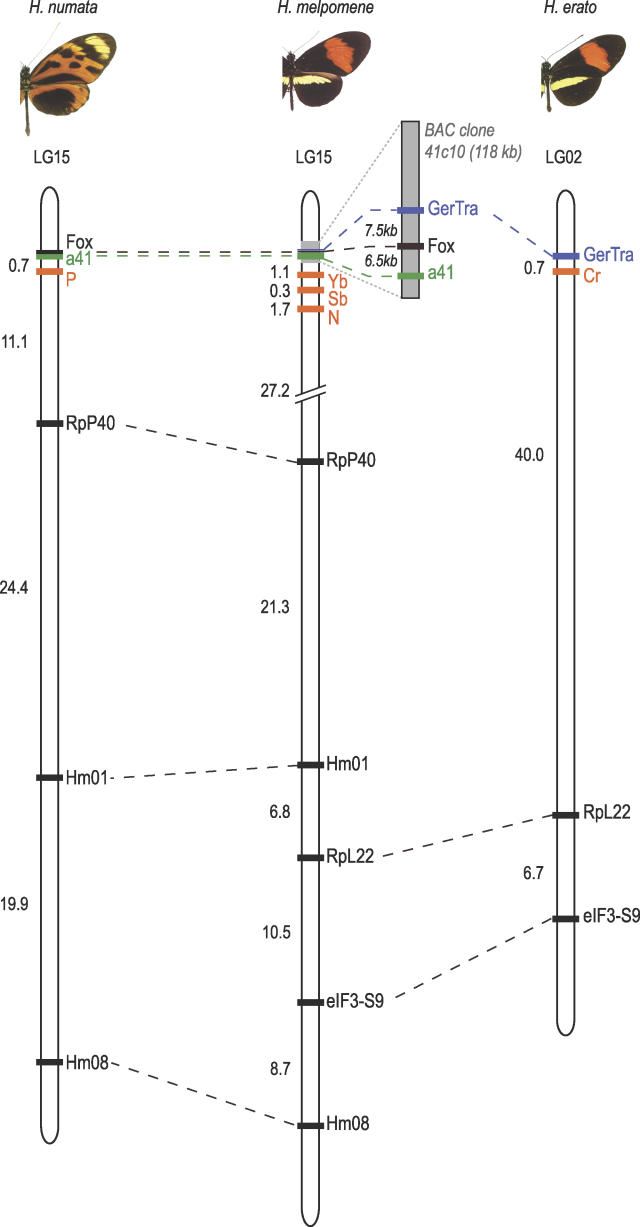
Chromosomal Maps for Linkage Group Homologues in H. melpomene (LG15), H. numata (LG15), and H. erato (LG02) Distances are in Haldane centimorgans. The alternative orders for *P* and *a41* relative to *Hm01* in H. numata are not significantly different (ΔLn*L* = −1.40). Similarly, most orders of *N, Sb, Yb,* and *a41* in H. melpomene are not significantly different (from ΔLn*L* = −0.15 for the order *a41–Yb*–*N–Sb–* to ΔLn*L* = −0.77 for *a41–N–Yb–Sb–*). Finally, the two orders for *Cr* and *GerTra* in H. erato are also equally significant. Therefore, we here show the most likely gene orders but cannot exclude that the colour loci are on the other side of the anchor loci *a41, Fox,* or *GerTra*. In contrast, anchor loci order *GerTra–RpP40–Hm01–Hm08* is robust, with alternative orders significantly worse (ΔLn*L* < −2), although the relative placement of *RpL22* and *eIF3*-*S9* is uncertain in H. melpomene and H. erato (ΔLn*L* > −2).

We demonstrated homology of the genomic location of the *P* locus in *H. numata,* the *N–Yb–Sb* complex in *H. melpomene,* and the *Cr* locus in H. erato (Figure 3). A noncoding region *(a41),* cloned from an amplified fragment length polymorphism marker in a linkage mapping study of *H. melpomene,* lies within 1.1 cM of the H. melpomene pattern locus *Yb* on linkage group 15 (out of a total map length of 1,616 cM) [22] (Figure S1). Among 413 individuals with both genotype and phenotype information from four mapping families, there were just five individuals recombinant between *a41* and *Yb* (Table S1). This same marker is located within 0.7 cM of the *P* locus, which controls polymorphism in *H. numata,* with only two recombinant individuals identified among 306 individuals derived from six mapping families (Table S2). The probability of finding *Yb* and *P* so tightly linked to a homologous marker in the two species by chance is *p* < 0.002 (see Materials and Methods).

**Figure 4:**
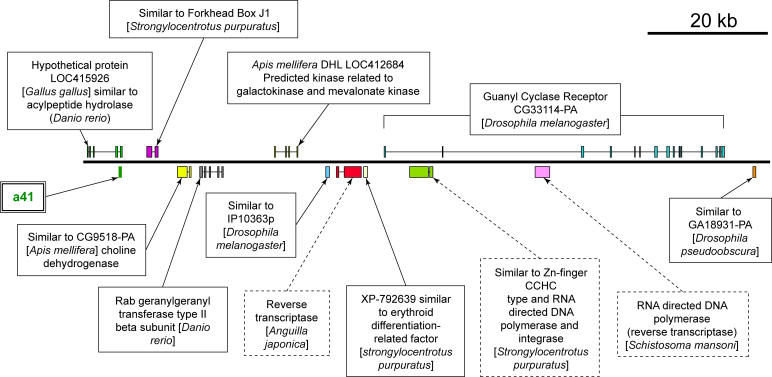
Annotation of Clone AEHM-41C10 from the Heliconius melpomene BAC Library The region is situated on LG15 in the H. melpomene genome [22]. The sequence contains open reading frames of strong homology to 12 reported genes, three of which appear to be retrotransposon-associated coding regions (dotted boxes). Also highlighted in double frames are the *a41* marker, which was used in H. numata and H. melpomene crosses and to isolate the clone from the library, and the Rabgeranylgeranyl transferase gene, used as a marker in H. erato crosses.

The primers for the noncoding *a41* marker did not amplify a product in H. erato. However, we used a PCR amplicon of this marker to probe a whole-genome bacterial artificial chromosomal (BAC) library of H. melpomene. A 118-kb BAC clone was identified and its genomic location confirmed by the following: (a) alignment with sequences of the *a41* locus generated from H. melpomene genomic DNA and (b) recombination mapping of at least one marker derived from the end sequences of this clone in both H. melpomene and H. numata. In both species, these end sequences showed complete linkage to *a41* in at least 100 individuals. This clone was then sequenced and annotated by BLAST comparison with nucleotide and protein sequence databases (see Materials and Methods; Figure 4). In addition to identifying the *a41* locus, we identified nine genes and three retrotransposon-associated coding regions (Figure 4).

None of the genes identified in the 118-kb BAC clone is a candidate for the *Yb* locus itself, because recombinants were identified between markers derived from the BAC end sequences and *Yb* in H. melpomene (unpublished data). However, coding sequences were used to design conserved PCR primers for gene-based markers that cross-amplify broadly across *Heliconius*. One of these markers, *GerTra,* amplifies using primers anchored in two putative exons of the *Rab geranylgeranyl transferase beta subunit (βggt-II)* gene and spans an intron showing substantial allelic size variation in *H. erato* (Figure S3). This region was 14 kb from the *a41* marker in H. melpomene (Figure 4), and variation at this locus segregated nearly perfectly with the colour locus *Cr* in *H. erato.* Only one recombinant between *Cr* and *GerTra* alleles was identified among 197 individuals from two mapping families of H. erato (Table S2), thus locating *GerTra* within 0.3 cM of the *Cr* locus (Figure 3; total map length in H. erato was estimated at 1,430 cM [27,28]). The probability of the H. melpomene gene *Yb* and H. erato gene *Cr* being tightly linked to homologous markers by chance is *p* < 0.003.

At a broader scale, two microsatellite markers *(Hm01* and *Hm08)* and three conserved gene regions *(eIF3-S9, RpL22,* and *RpP40)* map to the same linkage group as *Yb* in H. melpomene (Figure 2). In *H. numata, Hm01, Hm08,* and *RpP40* show a conserved pattern of linkage with H. melpomene both in terms of gene order and estimated distances between loci (Figure 2; *eIF3-S9* and *RpL22* were not variable in mapped broods of H. numata). The two microsatellite loci unfortunately do not cross-amplify in *H. erato,* but *RpL22* and *eIF3-S9* both map to the linkage group containing the *Cr* locus (Figure 2). These data reinforce our observation that linkage order is preserved between distantly related *Heliconius* species [27] and suggest that the chromosomes bearing colour genes *P, Yb,* and *Cr* have not undergone large-scale rearrangement between the three species.

In addition to genotyping *a41* and the markers derived from the BAC sequence, we have genotyped and assigned to linkage groups a total of 48 codominant molecular markers from across the genome, including 12 markers for genes known to be involved in the development of wings and patterns in other butterflies or in *Drosophila* (so-called candidate genes) [29–31], and 37 other conserved single-copy nuclear genes and microsatellites used as anchor loci in comparative mapping [22,27,28] (see Materials and Methods). We found no conflicting linkage relationship between the three species on the 16 linkage groups anchored with shared markers (Table 1) out of a total of 21 in each species [22,27], suggesting a widely conserved pattern of synteny at the genome scale. In *H. melpomene,* we have also mapped the following: (a) patterning loci *B* and *D,* which lie 66.7 cM from the gene *Cubitus-interruptus* on linkage group 18, (b) locus *Ac,* which is assigned to LG10, and (c) a locus we here term *Khw,* which lies 10 cM from the gene *wingless* on linkage group 1 (Table 1). *Khw* controls the white/yellow switch of the hindwing margins in *H. melpomene,* and it is putatively distinct from *K,* which controls the yellow/white switch of the forewing patch in H. cydno [23]. In both cases, the allele for white is dominant to that for yellow.

**Table 1:**
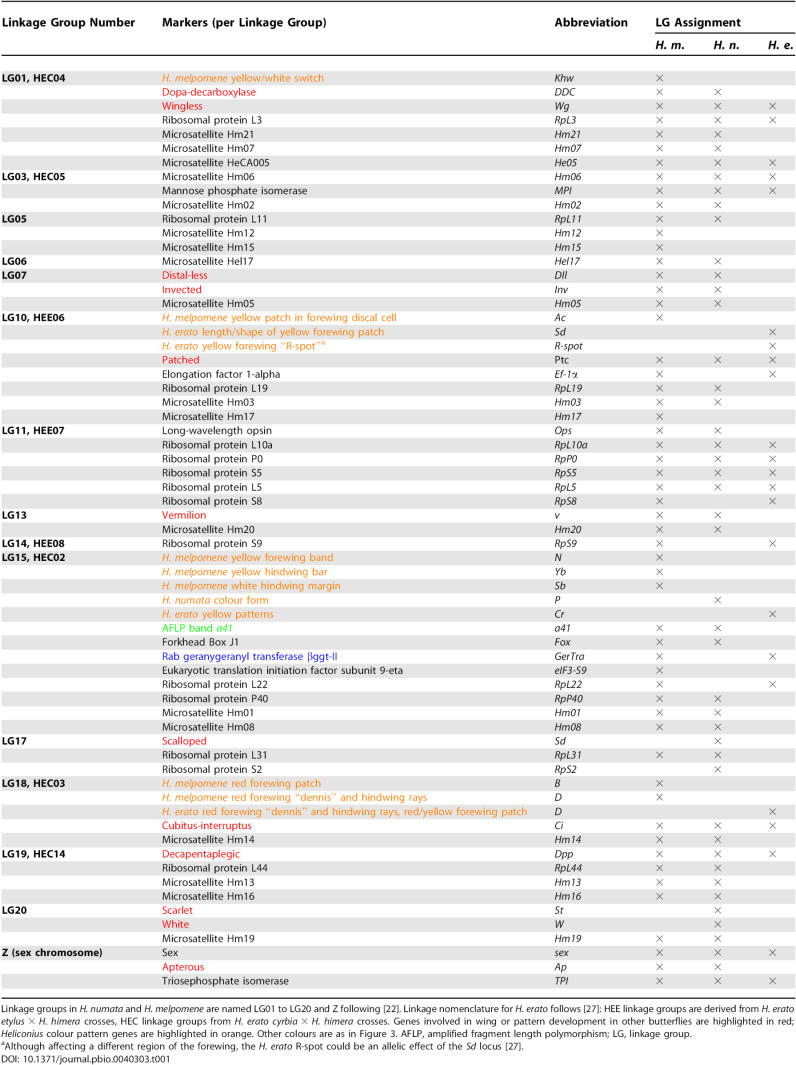
Linkage Group Associations in *H. melpomene, H. numata,* and *H. erato*

## Discussion

The data provide strong support for the hypothesis that a homologous gene or complex of genes regulates pattern diversity in *H. numata, H. melpomene,* and H. erato. The hypothesis of genetic homology of mimetic patterns in the geographic radiation of the Müllerian co-mimics H. erato and H. melpomene is a long-standing question, and our data provide the first explicit test, to our knowledge, of this hypothesis. It was initially suggested that shared developmental pathways might facilitate the convergence seen between mimetic species [5]. Subsequently, a more extreme hypothesis was proposed, which states that the actual genes (rather than merely pathways) might be homologous between species [16]. Here we have confirmed the hypothesis of homology, to under 10^−3^ of the *Heliconius* genome [22,27], of at least one of the major loci controlling convergent patterns between H. erato and H. melpomene (Figure 1).

Taken on its own, this result would apparently support the hypothesis that strong developmental constraints are important in mimicry evolution. Nonetheless, the positional genetic homology we demonstrate stands in striking contrast to the lack of colour pattern similarity [16] between the H. erato–H. melpomene pair and the patterns of *H. numata,* which are controlled by the same genomic region but involved in totally different mimicry rings (Figure 1). Rather than a constraint, this implies an extraordinary “jack-of-all-trades” flexibility of homologous colour pattern loci in closely related species (Figure 1). Our results in H. numata argue strongly against the idea that shared genetic architecture [8,32] constrains morphological diversification [7,33]. Instead, the data imply that natural selection has shaped a developmental switching mechanism capable of responding to a wide variety of mimetic pressures and producing locally adapted but highly divergent colour patterns.

The nature and mode of action for this developmental hot spot [34] of wing-pattern evolution remains to be determined. The tightly linked loci known to segregate in both the *H. melpomene N*–*Yb*–*Sb* complex and the *H. erato Cr* locus might represent a number of *cis* regulatory elements of a single switch gene [2,35], a cluster of duplicated genes with divergent function [35,36], or a cluster of nonparalogous but functionally related genes [37]. One or probably more of these distinct elements could be involved in the switch supergene of the H. numata polymorphic mimicry. The three tightly linked colour pattern loci *Yb, Sb,* and *N* clearly segregate on LG15 in *H. melpomene,* whereas *P (H. numata)* and *Cr (H. erato)* show only extremely rare recombinant phenotypes, which could reflect higher crossing-over rates in this genomic region in H. melpomene and/or the involvement of more genetic elements (Figure 3).

Colour patterns develop by the maturation and spatial arrangement of different types of scales on the surface of the developing wing, each characterised by specific pigments and cuticular ultrastructure [16,38]. Our data show that genes on many different chromosomes are involved in the development of the colour pattern. The yellow, red, and orange pigments in *Heliconius* are ommochromes, and the ommochrome pathway genes —*vermilion, white,* and *scarlet*— are all unlinked to patterning genes segregating in our crosses in at least one of the species (Table 1). Furthermore, signalling-pathway genes known to be involved in establishing spatial information in developing butterfly wings —such as *engrailed, Distal-less,* and *decapentaplegic* [29,30,39,40]—are also unlinked to switch genes. Two candidate genes were found to be linked to patterning loci *(Cubitus-interruptus* with *B* and *D,* and *wingless* with *Khw)*, but in both cases, recombination mapping ruled out a direct role for these loci (see below). We have shown that genes involved in wing development and pigment formation are distributed across the genome and not tightly linked to the patterning loci that we have mapped.

A number of observations from these and previous crosses, combined with the results obtained here, offer some clues as to the nature of the *N–Yb–Sb/Cr/P* complex. (a) The same pigment types are found in different genotypes at this locus in H. erato and H. melpomene (Figure 2B), demonstrating that these loci control placement of pigments but do not switch particular pigment pathways on or off constitutively. (b) In *H. melpomene,* several tightly linked loci control distinct pattern elements that can be separated by rare recombination. These loci have a similar function in that they all control placement of white or yellow pattern elements (Figure 2A), suggesting that they are either linked paralogous copies of the same gene, or clusters of *cis* regulatory elements of a single gene. (c) The locus controls patterns across both fore- and hindwings in all three species, but most strikingly in H. numata (Figure 2C). (d) The same allele can both increase and decrease the extent of the same pigment in different areas of the wing surface. In general, alleles adding yellow elements are recessive to those for black, but in the recessive *silvana* form of H. numata (allele *P^sil^,* Figure 2C), dominance of melanic elements is reversed relative to other forms such as *tarapotensis (P^tar^)*. Items (c) and (d) imply that the gene product(s) are not directly involved in determining spatial positioning across the wing but are more likely transcribed in response to spatial information. Therefore, this complex locus most likely acts by communicating between spatial coordinate pathways and pigment pathways to create colour pattern elements. We hypothesise that the switch gene is most likely a transcription factor with a number of *cis* regulatory elements that respond to the spatial information present in different parts of the wing. This transcription factor then triggers a response in sequentially acting downstream pathways to affect pigment deposition and scale morphology that are characteristic of each pattern element. Such regulatory elements would segregate in our crosses between wild forms and might vary in numbers and/or distance across species.

We have shown that another major mimicry locus lies on a homologous chromosome in the two co-mimics (Table 1). *Cubitus-interruptus* is 75 cM from *D* in H. erato [27] and 66.7 cM from *B* and *D* in H. melpomene (unpublished data). Given the loose linkage, a more precise positional comparison of these loci awaits fine-scale mapping of this linkage group, but the similarity of phenotypic effects of those loci and their location on homologous chromosomes hint at possible genetic homology of *B–D* and *D,* and, together with colour pattern loci on other linkage groups (Table 1), hint at a largely shared multilocus colour pattern architecture between the distantly related co-mimics H. erato and H. melpomene. Taken together, these findings in turn reveal a probable route for the evolution of the unusual “supergene” pattern control of H. numata. Local mimicry polymorphism in H. numata is stable and is associated with selection favouring single-locus control of the entire pattern *(P)* with hierarchical dominance and avoiding nonmimetic intermediates [19,20,41]. However, the evolution of such supergene architecture, where the cosegregation of wing characters can be broken up by recombination [19], and which is most widely known from polymorphic Batesian mimics such as Papilio dardanus or P. memnon [42–44], is a puzzle. Theory predicts that selection against nonmimetic recombinants will rarely lead to the evolution of closer linkage between unlinked elements [41]. Genes must be rather tightly linked in the first place [41,45–47]—for instance via local gene duplications or regulatory element expansion [35,36]—to provide a useful starting point for the evolution of tighter linkage. In contrast to that of *H. numata,* geographic radiation in H. melpomene is controlled by several unlinked regulatory loci of large effect *(N–Yb–Sb, B–D, Ac,* and *K),* and nonadaptive recombinants are probably not a focus of selection because they occur only in narrow hybrid zones [15,17,21]. More distantly related *Heliconius,* such as *H. erato,* also have a similar and probably largely homologous multichromosomal mimicry architecture [17,27,38] (Table 1), so that the single-locus inheritance in H. numata is a derived state (Figure 1). Our results thus suggest that part or all of the existing *N–Yb–Sb* complex of H. melpomene has evolved into *P* in *H. numata,* by taking control of regulation of the entire wing pattern [43,47], whereas the remaining unlinked colour pattern loci *(B, D, Ac,* and *K* in *H. melpomene; D* and *Sd* in *H. erato)* do not cosegregate with major colour pattern variation in H. numata (Table 1). This result provides the first empirical evidence against the hypothesis of a “supergene” evolving via a gradual tightening of linkage between previously loosely linked or unlinked genes; this hypothesis has previously been challenged only on theoretical grounds [41,45–47]. The elucidation of the mechanism by which *P* may have gained control of the entire regulation of wing pattern in H. numata will require the precise identification of the regulatory regions involved at this locus and the developmental pathways in which they take part [35].

To this end, the markers we developed provide a decisive step towards positional cloning of loci underlying colour pattern shifts. Our markers on LG15 are situated within a fraction of a centimorgan of the actual loci under selection, which may represent as little as 150 kb, given the estimated physical-to-map distance of ~165–180 kb/cM [22,28]. The genomic resources now available for positional cloning and large-scale sequencing in the three *Heliconius* species mean that we are now close to identifying the genes involved in this adaptive radiation [48]. Fine-scale mapping using densely distributed markers will locate the recombination breakpoints in our crosses and narrow the segregating locus to a region of a few kilobases. Furthermore, the phenotypes studied occur in the wild and segregate across natural hybrid zones or in polymorphic populations [17,18,20], which will facilitate the use of association studies to test candidate loci [8]. On a broader phylogenetic scale, the identification of the colour pattern alleles segregating in different forms, races, and species in the wild will allow insights into the history of variation at these major loci and lead to testable hypotheses regarding the historical, developmental, or genetic constraints underlying the repeated recruitment of alleles at specific genes in mimetic lineages. Unravelling the molecular structure and developmental role of this locus in *Heliconius* will therefore provide important insights into the evolutionary basis of adaptive novelty.

## Materials and Methods

### Crosses.

*H. melpomene cythera* (Mindo, Ecuador) and *H. m. malleti* (Rio Quijos, Baeza, Ecuador) were each crossed to a stock of *H. m. melpomene* (French Guiana) to generate F2 mapping families in Gamboa, Panama, following methods described previously [17]. The *Yb* and *Sb* loci were scored in 419 individuals from four replicate *H. m. cythera* × *H. m. melpomene* F2 crosses. *Yb* was scored as codominant based on the altered reflectance of heterozygote phenotypes [15], whereas *Sb* was considered dominant (Figure 2A). *N* was scored in 281 individuals from two *H. m. malleti* × *H. m. melpomene* F2 crosses (Table S1). Using a similar protocol, F2 and backcross families were derived from local forms of H. numata in Tarapoto, in eastern Peru [20]. Genotypes at the *P* locus in H. numata were scored in 306 individuals representing three F2 families of heterozygous *H. n.* f. *elegans (P^ele^/P^sil^)* fathers to *H. n.* f. *aurora (P^aur^/P^sil^)* or *H. n.* f. *arcuella (P^arc^/–)* mothers, and three backcrosses to homozygous *H. n.* f. *silvana (P^sil^/P^sil^)* mothers (Table S2; Figure 2C). In *H. erato,* the *Cr* locus was genotyped in a backcross (76 individuals) and an F2 intercross (117 individuals) between *H. e. cyrbia* (Guayquichuma Glen, Ecuador) and H. himera (Vilcabamba, Ecuador), generated in insectaries in Puerto Rico. Alternative alleles at the *Cr* locus are codominant in these crosses, although distinguishing *Cr^hi^Cr^c^* genotypes was more difficult in some genetic backgrounds [24,28] (Table S3; Figure 2B). In addition, a reference F2 intercross (97 individuals) between *H. e. notabilis* (Puyo, Ecuador) and H. himera (Vilcabamba, Ecuador) was genotyped for PCR markers *GerTra* and *RpL22;* this reference cross does not segregate for *Cr* (Table S3). Parents and progeny were either frozen at −80 °C or preserved in 20% dimethylsulphoxide, 0.25 M EDTA, and saturated NaCl solution (DMSO). DNA was extracted from thorax using the Qiagen DNeasy kit (Hilden, Germany) following manufacturer's instructions.

### Marker loci.

Development of most of the molecular markers we used is described elsewhere [22,49–51]. Specific primers for single-copy nuclear loci, such as ribosomal proteins, were developed from EST sequences, amplicon length variation and RFLPs were used to genotype segregating alleles in mapping families and PCR products were visualised on 1.5% agarose gels. Microsatellites were genotyped using fluorescent-labelled primers on an ABI 3730 capillary sequencing machine (Applied Biosystems, Foster City, California, United States). Specific primers were developed for the amplified fragment length polymorphism marker *a41,* previously identified as being linked to the *H. melpomene Yb* locus [21], to allow amplification of this locus in both H. numata and H. melpomene (Beltrán M, Mavárez J, González M, Bermingham E, Jiggins C, unpublished data). An alignment of H. melpomene and *H. numata a41* sequences is given in Figure S1. The *a41* region was amplified in H. numata and H. melpomene with a fluorescent-labelled primer, and the product was visualised as for microsatellite loci. In all broods except one, length variation segregated at the *a41* locus and could be scored as for microsatellites.

### Mapping.

Alleles derived from the mother (female-informative) were used to confirm synteny of linked markers [21], because chromosomes are inherited intact from the mother owing to the lack of crossing over in female Lepidoptera [52]. Alleles derived from the brood father (male-informative) were scored as for a backcross brood, and recombination distances were calculated using MapMaker [53]. Linkage group assignation was carried out using JoinMap 3.0 [54], and was based on the genotyping of brood Br33 in H. melpomene (148 individuals) [22] and broods B502 and B472 in H. numata (168 individuals). The probability of the markers being tightly linked to *a41* by chance was calculated as the probability of *Yb* and *P* being on the same chromosome (1/21) multiplied by the probability of *Yb* being within 1.1 cM of *a41* on the chromosome (2.2/56.0), conservatively using the H. numata linkage group 15 length of 56.0 map units.

### BAC clone identification and sequencing.

An H. melpomene BAC library was constructed by Amplicon Express (Pullman, Washington, United Sates) from high–molecular weight DNA derived from six larvae of H. melpomene. A total of 18,816 clones were picked, with an average insert size of 123 kb, giving an estimated 8× genome coverage. The entire library was gridded onto nylon membranes in a high-density 4 × 4 array of 6,144 (16 × 384) spots, each representing a single clone gridded once. These arrays were hybridised with a PCR-derived probe for the *a41* marker labelled with P32 using the Prime-It II Random Primer Labeling Kit (Stratagene, La Jolla, California, United States). Probe cleanup was carried out using a NucTrap Probe Purification Column (Stratagene). Hybridisation of the filters was carried out using protocols recommended by the Sanger Institute (http://www.sanger.ac.uk/HGP/methods/mapping/screening/hybs.shtml). A single positive clone was identified and confirmed by PCR. This clone was then sequenced, assembled, and finished by the Sanger Institute. Briefly, the clone was sheared to create 4- to 6-kb fragments that are cloned as a library into pUC19. Approximately 6× sequence coverage of each BAC was then generated in paired 600- to 800-bp reads. Data were assembled using Phrap software and edited in a GAP4 database. Contigs were extended by oligo walking. The BAC sequence was annotated using BLAST comparison with a UniRef100 database and with our *Heliconius* EST dataset.

### Development of the *GerTra* marker.

The following primers were then designed to span a 542-bp intron between two exons showing homology to the *Rab geranylgeranyl transferase beta subunit* (*βggt-II; Homo sapiens*) (Figure 4 and Figure S3): GerTra-Int-F 5′-ctgcgctgtgatgtgtcttt*-*3′ and GerTra-Int-R 5′-ggaggacattacccacctgt*-*3′. These primers amplified a single 1.2-kb product in *H. erato,* which was sequenced to confirm homology with the H. melpomene region (see Figure S2 for an alignment with the H. melpomene BAC clone sequence). New *H. erato–*specific primers (GerTra-Int-He-F 5′-ggctgttgattttgtgttaag-3′ and GerTra-Int-He-R 5′-attctgacatcaaaaagaggc-3′) were designed that gave more consistent amplification from genomic DNA. Genotypes at this locus were determined by following the segregation of allelic size variants on 1%–2% agarose gels.

## Supporting Information

Figure S1 Alignment of *a41* Sequences for H. melpomene and H. numata
The high homology of the sequences (scores > 84) confirms that the fragments represent orthologous markers in both species. The large insertions and deletions in the middle of the sequence allowed easy genotyping (Beltrán M, Mavárez J, González M, Bermingham E, Jiggins C, unpublished data).
(30 KB DOC)

Figure S2 Alignment of a *H. melpomene a41* Sequence with BAC Clone AEHM-41C10 The marker corresponds to positions 5,829–6,170 on the BAC sequence.
(27 KB DOC)

Figure S3 Alignment of *H. erato GerTra* Sequences with H. melpomene BAC Clone AEHM-41C10 Because the PCR amplicons in H. erato are too large for complete sequencing, we provide here the alignment of both end sequences with the respective *H. melpomene Rab geranylgeranyl transferase* exons from which the primers were designed. Exon 1 lies at position 19,970:20,290 and exon 2 at 21,535:21,846, with a 1,245-bp intron in between.
(38 KB DOC)

Table S1 Mapping Families and Colour Pattern Genotypes in H. melpomene
Details of the *H. m. cythera* × *H. m. melpomene* F2 crosses segregating for *Yb^c^/Yb, Sb^c^/Sb,* and *Khw^w^/Khw,* and *H. m. malleti* × *H. m. melpomene* F2 crosses segregating for *N^N^/N^B^, B/b,* and *D/d* (full pedigree information available upon request; codes in brackets identify the brood from which each parent originates). *Khw* is only expressed in a *Sb^c^/Sb^c^* background. See Figure 2A for details of wing patterns.
(98 KB DOC)

Table S2 Mapping Families and Colour Pattern Genotypes in H. erato
Details of the F2 cross (CH-CH5) and the backcross (CH-Cy6) of *H. e. cyrbia* × H. himera segregating for *Cr^cyr^/Cr^him^*. *Cr* alleles do not segregate in the NOTF2–9 reference F2 cross *H. e. notabilis* × *H. himera*, which was used to map gene markers *GerTra* and *RpL22*. Segregation at unlinked colour pattern loci *D* and *Sd* is given for reference. See Figure 2B for details of wing patterns.
(45 KB DOC)

Table S3 Mapping Families and Colour Pattern Genotypes in H. numata
Coloured superscript numbers identify chromosomes identical by descent in different broods (full pedigree information available upon request; codes in brackets give the brood from which each parent originates). See Figure 2C for details of wing patterns.
(70 KB DOC)

### Accession Numbers

The Genbank (http://www.ncbi.nlm.nih.gov) accession number for the H. melpomene BAC clone AEHM-41C10 is CR974474.

## Abbreviations

BAC: bacterial artificial chromosome
cM: centimorgan
