# Supplementary material for: A Conserved Supergene Locus Controls Colour Pattern Diversity in Heliconius Butterflies
Source: PLoS Biol. 2006 Sep 26;4(10):e303. doi: 10.1371/journal.pbio.0040303 (PMC1570757; doi:10.1371/journal.pbio.0040303)
Supplement: Supplementary file 1 [file pbio.0040303.sg001.doc]

**Figure S1. Alignment of *a41* sequences for *H. melpomene* and *H. numata*.**

The high homology of the sequences (scores > 84) confirms that the fragments represent orthologous markers in both species. The large insertions and deletions in the middle of the sequence allowed easy genotyping. From Beltrán *et al.* (submitted manuscript) Identification and phylogenetic history of a marker linked to a colour pattern gene in *Heliconius* *melpomene* (Ref. 25).

```
[ALGORITHM] CW
[MATRIX] IUB
[OPEN PENALTY] 10
[EXTEND PENALTY] 0.05
[EXTEND PENALTY 2] -2
[DA LENGTH] 100
[QUERY SEARC 4H] D
[CLUSTALW RESULTS]
CLUSTAL W (1.8) Multiple Sequence Alignments
Pairwise Alignment
Sequences (1:2) Aligned. Score:  86
Sequences (1:3) Aligned. Score:  84
Sequences (2:3) Aligned. Score:  88
Multiple Alignment
There are 2 groups
Group 1: Sequences:   2      Score:6858
Group 2: Sequences:   3      Score:6432
Alignment Score 6257

[BEGIN MULTIPLE ALIGNMENT]

m150_H.m.mocoa        -------------ACTATTACGCAAAAACTAGTGAACAGATTTACCTATATGAAATTTCA
8543_H.m.plesseni     ----------------------CAAAAACTAGTGAACAGATTTA---G-ATGAAATTTCA
N235_H.numata         CAGTTTTTGTTCCACTATTACGCAAAAACTAGTAAACAGATTTA---G-ATGAAATTTCA

m150_H.m.mocoa        CAG-CT-TA------TATATCAGAATAACACATATACGACAACAAGTTTAAGCAGGTAAA
8543_H.m.plesseni     CAGCTTATAAGTAAGTATATCAGAATAACACATATACTACAACAAGTTTAAGCAGGTAAA
N235_H.numata         CAG-TT-TA--TAAGTATATCAGAATAACACATATACTACAACAAGTTTAAGCAGGTAAA

m150_H.m.mocoa        TCCGCATTTCGGTCGCTAGT--TCTA-AATA-TGATA--C--AAT--TTTATTTTCTT-A
8543_H.m.plesseni     TCCGCATTTCGGTCGCTAGTATGATACAAT-TTCATATTCTTAATACTCCATCATCTTTA
N235_H.numata         KCAGCATTTCGGTTGCTAGT--KCTA-AATACTC-TA--CTTAATACTCCRTCATCTTTA

m150_H.m.mocoa        -ATTTT--CATAATTTAAGCTTTTTTATTATAGCTGTTCGTGTATACATTTTTAAAGCTT
8543_H.m.plesseni     TGTTTTCCCATTGTTTAAGCTTTTTTATTATAGCTGTTCGTGTATACATTTTTAAAGCTT
N235_H.numata         TGTTTTTCCATAGTTTAAGCCTCTTTATTACAGCTGTTCGTGTATACATTTATAAAGCTT

m150_H.m.mocoa        ACATGATT-AT-G----A-TA-CT--T---GTTCTTGCCTTGTTTATTAAAATTTTTACC
8543_H.m.plesseni     ACATGATTTATAGTTTCAGTATCTAATAAAGTTCTTGCGTTGTTTATTAAAATTTTTACC
N235_H.numata         ACATGATTTATAGTTTCGGTATCAAATAAAGTTCTTGCCTTGTTTATAAAAATTTTTACT

m150_H.m.mocoa        CTTTATTAAGAATACTAATAATAACTTATAATTATTTTTCGACTTTTTTT-CAAAATATA
8543_H.m.plesseni     CTTTATTAAAAATACTAATAATAACTTATAATTCTTATTCGATTTTTTTTTCAAAATATA
N235_H.numata         CTTTATTAAAAAAACTAAAAATAACTTATAATTCTTTTTCTATTTTTTTTTCAAAATATA

m150_H.m.mocoa        AGATTTTTCTACATCTCCACAGGTGTTCAATAGAAGCTGGCATATCTTTCGAAGACGGTG
8543_H.m.plesseni     AGATTTTTCTACATCTCCGCAGGTGTTCAATAGAAGCTGGTATATCTTTCGTAGACG---
N235_H.numata         AGGTTTTTTTACATCTCCACAGGGGTTCAATAGAAGCTGGTATATCTTTCGTAGAC----

m150_H.m.mocoa        GTCAGGA
8543_H.m.plesseni     -------
N235_H.numata         -------
```

[END MULTIPLE ALIGNMENT]
