# Supplementary material for: A Conserved Supergene Locus Controls Colour Pattern Diversity in Heliconius Butterflies
Source: PLoS Biol. 2006 Sep 26;4(10):e303. doi: 10.1371/journal.pbio.0040303 (PMC1570757; doi:10.1371/journal.pbio.0040303)
Supplement: Supplementary file 2 [file pbio.0040303.sg002.doc]

**Figure S2. Alignment of a *H. melpomene a41* sequence with BAC clone AEHM-41C10.** The marker corresponds to positions 5829-6170 on the BAC sequences (Accession number CR974474)

```
#NEXUS

[!This data assembled using CodonCode Aligner.]
begin data;
	dimensions ntax=2 nchar=342;
	format datatype=dna gap=:  interleave;
	matrix

Hm-rosina-a41  CTYAGATGAAATTTCACAGCTTATAAGTATATCAGAATAACACATATT
BAC-5829-6170  cttagatgaaatttcacagcttataagtatatcagaataacacatatt

Hm-rosina-a41  CTACAACAAGTTTAAGCAAGTAAATCCACATTTCGGTCGCTAGTTCTA
BAC-5829-6170  ctacaacaagtttaagcaagtaaatccacatttcggtcgctagttcta

Hm-rosina-a41  AATATGATACAATTTTATTTTCTTAATTTTCATAATTTAAGCTYTTTT
BAC-5829-6170  aatatgatacaattttattttcttaattttcataatttaagctttttt

Hm-rosina-a41  ATTATAGCTGTTCGTGTATACATTTTTAAAGCTTACATGATTATGATA
BAC-5829-6170  attatagctgttcgtgtatacatttttaaagcttacatgattatgata

Hm-rosina-a41  CTTGTTCTTGCCTTGTTTATTAAAATTTTTACCCTTTATTAAGAATAC
BAC-5829-6170  cttgttcttgccttgtttattaaaatttttaccctttattaagaatac

Hm-rosina-a41  TAATAATAACTTATAATTATTTTTCGACTTTTTTTCAAAATATAAAAT
BAC-5829-6170  taataataacttataattacttttcgactttttttcaaaatataaggt

Hm-rosina-a41  TTTTCTACATCTCCACAGGTGTTCAATAGAAGCTGGNATATCTTTCGT
BAC-5829-6170  ttttttacatctccacaggtgttcaatagaagctggtatatctttcgt

Hm-rosina-a41  AGACGG
BAC-5829-6170  agacgg

;

endblock;
```
