# Supplementary material for: A Conserved Supergene Locus Controls Colour Pattern Diversity in Heliconius Butterflies
Source: PLoS Biol. 2006 Sep 26;4(10):e303. doi: 10.1371/journal.pbio.0040303 (PMC1570757; doi:10.1371/journal.pbio.0040303)
Supplement: Supplementary file 3 [file pbio.0040303.sg003.doc]

**Figure S3. Alignment of *H. erato* *GerTra* sequences with *H. melpomene* BAC clone AEHM-41C10.** The PCR amplicons in *H. erato* being too large for complete sequencing, we provide here the alignment of both end sequences with the respective *H. melpomene* Rab geranylgeranyl transferase exons from which the primers were designed. Exon 1 lies at position 19970:20290, and exon 2 at 21535:21846, with a 1245bp intron in between.

```
#NEXUS

[!This data assembled using CodonCode Aligner.]
begin data;
	dimensions ntax=2 nchar=652;
	format datatype=dna gap=:  interleave;
	matrix

Exon 1

H. melp. GerTra exon 1  ttatacttgtaaaatctgtggttctaacttcaacctatcaatggtttc
H. erato GerTra 1       ------------------------------------------------

H. melp. GerTra exon 1  ttgcggcatacaataagtagggttaacttgcttaatagtagagttccc
H. erato GerTra 1       ------------------------------------------------

H. melp. GerTra exon 1  taataaagatagtccggctaaaccgaataacgtgtggaaaggatcagt
H. erato GerTra 1       ------------------------------------------------

H. melp. GerTra exon 1  tatgtctccgggacggtcactgaaaccaccagtttcagaatcttgaca
H. erato GerTra 1       ------------------------------------------------

H. melp. GerTra exon 1  cgccagtatatattcttctagatttttcttgtcgacccagtgtattct
H. erato GerTra 1       ------------------------------------------------

H. melp. GerTra exon 1  gtttagcattgatagggaggacattacccacctgtaatatacatt---
H. erato GerTra 1       ---------------GGAGGACATTACCCACCTGTAATATAAATTAGA

H. melp. GerTra exon 1  ------------------------------------------------
H. erato GerTra 1       TTTTATCGTATTAATTCATTTATAATAGTAAGATTTAAAATAAATTTA

H. melp. GerTra exon 1  ------------------------------------------------
H. erato GerTra 1       GTTATAATATCCTATTTAATGAAGAATATTTTGTAATGTATTATTTTT

H. melp. GerTra exon 1  ------------------------------------------------
H. erato GerTra 1       TATTCTGACATCAAAAAGAGGCACATAATTTAAAATGTTTTTTATATA

H. melp. GerTra exon 1  ------------------------------------------------
H. erato GerTra 1       TACAAATATTTTATAATTTCCTTAATTTTATATAGAAATGTTTAACAT

H. melp. GerTra exon 1  ------------------------------------------------
H. erato GerTra 1       TCTAATAAAGCAGTAAGTGAGTCCATCTTACTTGGAAACTATAATAAT

H. melp. GerTra exon 1  ------------------------------------------------
H. erato GerTra 1       GTACAGTGCTAGAGATATTGGATACTTAATTTCTGCTAGGTGTATGTA

H. melp. GerTra exon 1  ------------------------------------------------
H. erato GerTra 1       ATATAATTAAGGCCGTCGTGGCAAAGTGGGGTAGCATCTCACTGTAAG

H. melp. GerTra exon 1  ----------------------------
H. erato GerTra 1       CGCGACGATTATAGGTTCAATCCTCACG


Exon 2.

H. melp. GerTra exon 2  ------------------------------------------------
H. erato GerTra 2       TTACCAAGAGAAAACACGTGGAGCTACAACAAATTGTAGCAGGAAAGT

H. melp. GerTra exon 2  ------------------------------------------------
H. erato GerTra 2       GCTTTGGATGATTGAGTTATTTATTAAAATTGCTAACCTTTTTTTATT

H. melp. GerTra exon 2  ------------------------------------------------
H. erato GerTra 2       AACATCTTAAAAAGTCATATTTTTAGACAATTTTAAACGAGGTAGTAG

H. melp. GerTra exon 2  ------------------------------------------------
H. erato GerTra 2       CGCCATAAGTGTGCGACTGTAAACGCGTAGGCTATGGGTTCGATTCTT

H. melp. GerTra exon 2  ------------------------------------------------
H. erato GerTra 2       ATTCGGGAGAATGAAATATTTAATATTTTATTCGGTCTTGTAACAGGA

H. melp. GerTra exon 2  ------------------------------------------------
H. erato GerTra 2       CAAAGTGCGGCATTGATTCCCACCACTAAGCATAGACAATCTCGACAA

H. melp. GerTra exon 2  ------------------------------------------------
H. erato GerTra 2       TTCGGCAGAAAGTGAATATGCGATATTTCGAGATATACTCGAAAGTTC

H. melp. GerTra exon 2  ------------------------------------------------
H. erato GerTra 2       AGTGGTATATTAACAGTGGGTTCCCGCTGCTTACCCCTGCTGTATGCG

H. melp. GerTra exon 2  ------------------------------------------------
H. erato GerTra 2       TGTTATAGCGTGATAGCTTAGTTTATAAAAAAATACAATTAAGCAAAA

H. melp. GerTra exon 2  -AATACCTGCATGACTTTCAGAACCAGGCTTAGATCCAAAACCTCCAT
H. erato GerTra 2       TAATACCCGCGTGACTCTCAGAACCAGGTTTAGATCCAAAACCGCCAT

H. melp. GerTra exon 2  CAAAATTCATACAGCTTAATACAAAATCAACAG-CTTTAGTTACATTT
H. erato GerTra 2       CAAAATTCATACAGCTTAACACAAAATCAACAGCCCTTGGTTACATTG

H. melp. GerTra exon 2  ATGGAGTCCAATTTATGCAATAATGAAAGACACATCACAGCGCAGAAA
H. erato GerTra 2       ATTGAGTACAACTTATGCAATAATGAAAGACACATCACAGCGCAG---

H. melp. GerTra exon 2  GAAAATCTTGTGTCAACTTCACCCCACTTGTCACCTAAGAAATTGAAA
H. erato GerTra 2       ------------------------------------------------

H. melp. GerTra exon 2  TTATTATATGTATTGATATTTATACCTTGTTTAGAAGACATTTTTTTT
H. erato GerTra 2       ------------------------------------------------

H. melp. GerTra exon 2  AATGTAAAGGGATTCAGTTGTCCAAGGGCTCTGATAACTAGGTTACAA
H. erato GerTra 2       ------------------------------------------------

H. melp. GerTra exon 2  CTCAATATTTTCTTAACACC
H. erato GerTra 2       --------------------


endblock;
```
