# Supplementary material for: A Conserved Supergene Locus Controls Colour Pattern Diversity in Heliconius Butterflies
Source: PLoS Biol. 2006 Sep 26;4(10):e303. doi: 10.1371/journal.pbio.0040303 (PMC1570757; doi:10.1371/journal.pbio.0040303)
Supplement: Supplementary file 4 [file pbio.0040303.st001.doc]

**Table S1. Mapping families and colour-pattern genotypes in *H. melpomene*.** Details of the *H. m. cythera* x *H. m. melpomene* F2 crosses segregating for *Yb^c^/Yb*, *Sb^c^/Sb*, and *Khw^w^/Khw*, and *H. m.* *malleti* x *H. m. melpomene* F2 crosses segregating for *N^N^/N^B^*, *B/b* and *D/d*. (Full pedigree information available upon request; codes in brackets identify the brood from which each parent originates). *Khw* is only expressed in a *Sb^c^/Sb^c^* background. See Figure 2a for details of wing patterns.

| **Brood details** | **Colour genotype** | | **Males** | **Females** | **Total** |
| --- | --- | --- | --- | --- | --- |
| *Brood 33* |  | |  |  |  |
| ♀ 10545 (B008) | *Yb^c^Yb, Sb^c^Sb* | |  | X |  |
| × ♂ 8261 (B002) | *Yb^c^Yb, Sb^c^Sb* | | X |  |  |
| Progeny: | *Yb^c^Yb^c^, Sb^c^Sb^c^, Khw^w^-* | | 11 | 15 | 26 |
|  | *Yb^c^Yb^c^, Sb^c^Sb^c^, Khw^Y^Khw^Y^* | | 7 | 3 | 10 |
|  | *Yb^c^Yb, Sb-* | | 33 | 39 | 72 |
|  | *YbYb, Sb-* | | 9 | 23 | 32 |
|  | *Yb-, Sb-* | | 3 | 4 | 7 |
|  | *Yb^c^Yb^c^, Sb-* | |  | 1 | 1 |
| *Brood 36* |  | |  |  |  |
| ♀ 11005 (B008) | *Yb^c^Yb, Sb^c^Sb* | |  | X |  |
| × ♂ 8290 (B002) | *Yb^c^Yb, Sb^c^Sb* | | X |  |  |
| Progeny: | *Yb^c^Yb^c^, Sb^c^Sb^c^, Khw^w^-* | | 9 | 13 | 22 |
|  | *Yb^c^Yb^c^, Sb^c^Sb^c^, Khw^Y^Khw^Y^* | | 2 | 1 | 3 |
|  | *Yb^c^Yb, Sb-* | | 15 | 25 | 40 |
|  | *YbYb, Sb-* | | 5 | 10 | 15 |
|  | *Yb-, Sb-* | | 3 | 3 | 6 |
| *Brood 43* |  | |  |  |  |
| ♀ 10781 (B008) | *Yb^c^Yb, Sb^c^Sb* | |  | X |  |
| × ♂ 10045 (B002) | *Yb^c^Yb, Sb^c^Sb* | | X |  |  |
| Progeny: | *Yb^c^Yb^c^, Sb^c^Sb^c^, Khw^w^-* | | 10 | 6 | 16 |
|  | *Yb^c^Yb^c^, Sb^c^Sb^c^, Khw^Y^Khw^Y^* | | 3 | 5 | 8 |
|  | *Yb^c^Yb, Sb-* | | 24 | 25 | 49 |
|  | *YbYb, Sb-* | | 14 | 13 | 27 |
|  | *Yb-, Sb-* | | 12 | 9 | 21 |
| *Brood 55* |  | |  |  |  |
| ♀ 10880 (B008) | *Yb^c^Yb, Sb^c^Sb* | |  | X |  |
| × ♂ 10095 (B002) | *Yb^c^Yb, Sb^c^Sb* | | X |  |  |
| Progeny: | *Yb^c^Yb^c^, Sb^c^Sb^c^, Khw^w^-* | | 2 | 8 | 10 |
|  | *Yb^c^Yb^c^, Sb^c^Sb^c^, Khw^Y^Khw^Y^* | | 0 | 5 | 5 |
|  | *Yb^c^Yb, Sb-* | | 5 | 14 | 19 |
|  | *YbYb, Sb-* | | 6 | 16 | 22 |
|  | *Yb-, Sb-* | | 3 | 5 | 8 |
| *Total Yb, Sb and Khw segregation* | |  |  |  | 419 |
|  |  | |  |  |  |
| *Brood 44* |  | |  |  |  |
| ♀ 11172 (B016) | *N^B^N^N^* | |  | X |  |
| × ♂ 10047 (B009) | *N^B^N^N^* | | X |  |  |
| Progeny: | *N^N^N^N^* | | 17 | 26 | 43 |
|  | *N^B^N^N^* | | 38 | 46 | 84 |
|  | *N^B^N^B^* | | 16 | 25 | 41 |
| *Brood 48* |  | |  |  |  |
| 11021 (B016) |  | |  | X |  |
| 10055 (B021) |  | | X |  |  |
| Progeny: | *N^N^N^N^* | | 14 | 21 | 35 |
|  | *N^B^N^N^* | | 27 | 25 | 52 |
|  | *N^B^N^B^* | | 12 | 14 | 26 |
| *Total N segregation* |  | |  |  | 281 |
|  |  | |  |  |  |
| *Brood 44* |  | |  |  |  |
| ♀ 11172 (B016) | *Bb, Dd* | |  | X |  |
| × ♂ 10047 (B009) | *Bb, Dd* | | X |  |  |
| Progeny: | *B-, D-* | | 44 | 46 | 90 |
|  | *bb, D-* | | 14 | 30 | 44 |
|  | *B-, dd* | | 16 | 23 | 39 |
| *Brood 48* |  | |  |  |  |
| 11021 (B016) | *Bb, Dd* | |  | X |  |
| 10055 (B021) | *Bb, Dd* | | X |  |  |
| Progeny: | *B-, D-* | | 30 (2) | 38 | 68 |
|  | *bb, D-* | | 12 | 17 | 29 |
|  | *B-, dd* | | 12 | 8 | 20 |
| *Total B and D segregation* | |  |  |  | 290 |
