# Supplementary material for: A Conserved Supergene Locus Controls Colour Pattern Diversity in Heliconius Butterflies
Source: PLoS Biol. 2006 Sep 26;4(10):e303. doi: 10.1371/journal.pbio.0040303 (PMC1570757; doi:10.1371/journal.pbio.0040303)
Supplement: Supplementary file 5 [file pbio.0040303.st002.doc]

**Table S2. Mapping families and colour-pattern genotypes in *H. erato*.** Details of the F2 cross (CH-CH5) and the backcross (CH-Cy6) of *H. e. cyrbia* x *H. himera* segregating for *Cr_cyr_/Cr_him_*. *Cr* alleles do not segregate in the NOTF2-9 reference F2 cross *H. e. notabilis* x *H. himera* for, which was used to map gene markers *Ger-Tra* and *RpL22*. Segregation at unlinked colour-pattern loci *D* and *Sd* is given for information. See Figure 2b for details of wing patterns.

| **Brood details** | **Colour genotype** | **Males** | **Females** | **Total** |
| --- | --- | --- | --- | --- |
| *Brood CH-CH5* |  |  |  |  |
| ♀ 2000- PR230 | *Cr^hi^Cr^cy^ D^hi^D^cy^* |  | X |  |
| × ♂ 2000- PR253 | *Cr^hi^Cr^cy^ D^hi^D^cy^* | X |  |  |
| Progeny: | *Cr^hi^Cr^hi^ D^hi^D^cy^ Cr^hi^Cr^hi^ D^hi^D^hi^*  *Cr^hi^Cr^hi^ D^cy^D^cy^* | 17 | 14 | 31 |
|  | *Cr^cy^Cr^cy^ D^hi^D^cy^ Cr^cy^Cr^cy^ D^hi^D^hi^*  *Cr^cy^Cr^cy^ D^cy^D^cy^* | 13 | 15 | 28 |
|  | *Cr^hi^Cr^cy^ D^hi^D^cy^ D^hi^D^hi^ Cr^hi^Cr^cy^*  *D^cy^D^cy^ Cr^hi^Cr^cy^* | 21 | 37 | 58 |
| *Brood CH-Cy6* |  |  |  |  |
| ♀ 2000- PR210 | *Cr^cy^Cr^cy^ D^cy^D^cy^* |  | X |  |
| × ♂ 2000- PR130 | *Cr^hi^Cr^cy^ D^hi^D^cy^* | X |  |  |
| Progeny: | *Cr^cy^Cr^cy^ D^cy^D^cy^ Cr^cy^Cr^cy^ D^hi^D^cy^* | 16 | 9 | 25 |
|  | *Cr^hi^Cr^cy^ D^cy^D^cy^ Cr^hi^Cr^cy^ D^hi^D^cy^* | 23 | 28 | 51 |
| *Total Cr segregation* |  | *90* | *93* | *183* |
|  |  |  |  |  |
| *Brood NOTF2-9* |  |  |  |  |
| ♀ 2000- PR280 | *Sd^hi^Sd^not^ D^hi^D^not^* |  | X |  |
| × ♂ 2000- PR247 | *Sd^hi^Sd^not^ D^hi^D^not^* | X |  |  |
| *Total:* |  | *54* | *43* | *97* |
| *Total reference brood* |  | 144 | 146 | 290 |
