# Supplementary material for: A Conserved Supergene Locus Controls Colour Pattern Diversity in Heliconius Butterflies
Source: PLoS Biol. 2006 Sep 26;4(10):e303. doi: 10.1371/journal.pbio.0040303 (PMC1570757; doi:10.1371/journal.pbio.0040303)
Supplement: Supplementary file 6 [file pbio.0040303.st003.doc]

**Table S3. Mapping families and colour-pattern genotypes in *H. numata*.** Coloured **s**uperscript numbers identify chromosomes identical by descent in different broods (full pedigree information available upon request; codes in brackets give the brood from which each parent originates). See Figure 2c for details of wing patterns.

| **Brood details** | **Colour genotype** | **Males** | **Females** | **Total** |
| --- | --- | --- | --- | --- |
| *Brood B 502* |  |  |  |  |
| ♀ 2002-502 (B390) | *P^aur^*^(^**^1^**^)^*P^sil^* |  | X |  |
| × ♂ 2002-425 (B319) | *P^ele^*^(^**^2^**^)^*P^sil^*^(^**^3^**^)^ | X |  |  |
| Progeny: | *P^ele^P^aur^* | 13 | 8 | 21 |
|  | *P^ele^P^sil^* | 12 | 6 | 18 |
|  | *P^sil^P^aur^* | 14 | 18 | 32 |
|  | *P^sil^P^sil^* | 10 | 7 | 17 |
| *Brood B 472* |  |  |  |  |
| ♀ 2002-472 (B390) | *P^sil^P^sil^* |  | X |  |
| × ♂ 2002-421 (B319) | *P^ele^*^(^**^2^**^)^*P^sil^* | X |  |  |
| Progeny: | *P^ele^P^sil^* | 24 | 21 | 45 |
|  | *P^sil^P^sil^* | 19 | 16 | 35 |
| *Brood B 511* |  |  |  |  |
| ♀ 2002-511 (B383) | *P^sil^P^sil^* |  | X |  |
| × ♂ 2002-425 (B319) | *P^ele^*^(^**^2^**^)^*P^sil^*^(^**^3^**^)^ | X |  |  |
| Progeny: | *P^ele^P^sil^* | 7 | 10 | 17 |
|  | *P^sil^P^sil^* | 9 | 13 | 22 |
| *Brood B 461* |  |  |  |  |
| ♀ 2002-461 (B332) | *P^arc^*^(^**^4^**^)^*P^aur^*^(^**^1^**^)^ |  | X |  |
| × ♂ 2002-425 (B319) | *P^ele^*^(^**^2^**^)^*P^sil^*^(^**^3^**^)^ | X |  |  |
| Progeny: | *P^ele^P^arc^* | 5 | 4 | 9 |
|  | *P^ele^P^aur^* | 6 | 3 | 9 |
|  | *P^sil^P^arc^* | 5 | 5 | 10 |
|  | *P^sil^P^aur^* | 6 | 2 | 8 |
|  | *P^ele^*– | 1 | 1 | 2 |
| *Brood B 446* |  |  |  |  |
| ♀ 2002-446 (B332) | *P^arc^*^(^**^4^**^)^*P^sil^* |  | X |  |
| × ♂ 2002-424 (B319) | *P^ele^*^(^**^2^**^)^*P^sil^*^(^**^3^**^)^ | X |  |  |
| Progeny: | *P^ele^P^arc^* | 3 | 5 | 8 |
|  | *P^ele^P^sil^* | 2 | 5 | 7 |
|  | *P^sil^P^arc^* | 6 | 4 | 10 |
|  | *P^sil^P^sil^* | 4 | 1 | 5 |
|  | *P^sil^*– | 1 |  | 1 |
| *Brood B 523* |  |  |  |  |
| ♀ 2002-523 (B383) | *P^sil^P^sil^* |  | X |  |
| × ♂ 2002-455 (B332) | *P^arc^*^(^**^4^**^)^*P^aur^*^(^**^1^**^)^ | X |  |  |
| Progeny: | *P^arc^P^sil^* | 9 | 6 | 15 |
|  | *P^aur^P^sil^* | 9 | 6 | 15 |
| *Total* |  | 162 | 144 | 306 |
